# One-year outcome and quality of life of patients with subarachnoid hemorrhage admitted to intensive care unit: a single-center retrospective pilot study

**DOI:** 10.1186/s44158-024-00223-w

**Published:** 2025-01-03

**Authors:** Carlo Bergamini, Etrusca Brogi, Sara Salvigni, Cesena Outcome Group, Diletta Castagnoli, Mattea Catucci, Chiara Gasperini, Erica Cecchini, Moana Bevoni, Sabina Bartoletti, Maria Russo, Serena Faedo, Dario Pietrantozzi, Silvia Passero, Michele Romoli, Giovanni Bini, Alessandra Venditto, Elvis Lafe, Marcello D’Andrea, Luigino Tosatto, Maria Ruggiero, Vanni Agnoletti, Emanuele Russo

**Affiliations:** 1 Department of Emergency Surgery and Trauma, Anesthesia and Intensive Care Unit, Bufalini Hospital, Azienda Unità Sanitaria Locale (AUSL) Della Romagna, Cesena, Italy; 2 Neuroscience Intensive Care Unit, ASST Grande Ospedale Metropolitano Niguarda, Milan, Italy; 3 Neurology and Stroke Unit, Department of Neuroscience, Bufalini Hospital, Cesena, Italy; 4 Neuroradiology, Department of Neuroscience, Bufalini Hospital, Cesena, Italy; 5 Department of Neurosurgery, Maurizio Bufalini Hospital, Cesena, Italy

**Keywords:** Subarachnoid hemorrhage, Aneurysmal subarachnoid hemorrhage, Intensive care, Outcome, Prognosis

## Abstract

Patients admitted to intensive care unit (ICU) after non-traumatic subarachnoid hemorrhage (SAH) represent a group with distinctive characteristics and few data are available on long-term outcome in this population. We conducted a single-center retrospective study in an Italian intensive care unit. All patients with non-traumatic SAH (ICD-9-CM Diagnosis Code 430) admitted to ICU were included. Disability and quality of life were evaluated via telephone interview after 12–15 months after initial bleeding using GOSE and EuroQoL, respectively. Baseline and clinical course characteristics were analyzed to evaluate relation with poor outcome defined as GOSE ≤ 3. Final population consisted of 38 patients. Twenty-four patients (63.2%) had favorable outcome (GOSE ≥ 4). Among 29 patients (76.3%) who survived at 1 year, median EQ-5D Index was 0.743 (IQR 0.287), while median EQ-VAS was 74.79 (IQR 18.5). Median EQ-5D Index and median EQ-VAS were higher among patients with favorable outcome (EQ-5D Index *p* = 0.037, EQ-VAS *p* = 0.003). Among baseline characteristics, only HH scale showed a significant relation with disability at one year (*p* = 0.033). Between complications occurred during ICU-stay only early HICP was related with unfavorable outcome (*p* = 0.028). Higher HH scale and early HICP were related with unfavorable outcome. Among patients with unfavorable outcome, quality of life has a broad range of variability, and this result should be taken into account when reporting patient-centered outcomes.

## Introduction

Non-traumatic subarachnoid hemorrhage (SAH) is a life-threatening emergency caused by bleeding in the subarachnoid space caused mainly by a ruptured aneurysm [1]. Despite recent advances in subarachnoid hemorrhage treatment, overall mortality and severe disability remain high, and this represents a major public health concern since the population affected is younger compared with other kinds of stroke. As shown in the Swiss SOS study, of all patients affected by SAH, 1 in 10 patients survived in a dependent state at 1 year, while 22% of patients died within 1 year [2]. Furthermore, among SAH survivors with good functional outcomes, quality of life is often impaired.

The pathophysiology of brain injury in SAH patients is extremely complex and not fully understood. The main determinants of brain injury are early brain injury (EBI) and delayed cerebral ischemia (DCI) [3]. EBI encompasses several disorders occurring within the first 72 h following the aneurysm rupture. Extravasation of blood into the subarachnoid space causes an increase in intracranial pressure (ICP) and consequent reduction of cerebral blood flow and transient global cerebral ischemia. Moreover, hydrocephalus could represent a life-threatening emergency in the acute phase and could aggravate the injury. Other mechanisms leading to poor outcome are vasospasm and DCI [3]. Even though cerebral vasospasm has been considered the main determinant of DCI, recent data show that pathophysiology is far more complex than once thought; in fact, cortical spreading depression, microthrombosis, and impaired collateral circulation could contribute to DCI and consequent brain tissue infarction [4].

Predicting outcomes among critical care patients is difficult: Advances in medicine have reduced short-term mortality from critical illness despite an increasing number of older patients, but this poses many questions about the cost of survival. As widely reported, patients surviving critical illness develop long-lasting impairments affecting physical, cognitive, and/or mental health status [5]. Furthermore, among neurocritical care patients, the burden of injury can be significant on long-term outcomes, and this should be considered since the dichotomy survived/death cannot fully describe the impact of the disease on patients. Long-term outcome in SAH is mainly inferred from randomized control trial cohorts, and even fewer data are available from patients with poor grade SAH admitted to intensive care unit (ICU). A recent French study showed as only two-thirds of patients survive at one year, and only one-third of them has a good outcome [6].

As highlighted by several organizations, including WHO, disability is just a partial description of the health status of a patient [7]; the patients’ experiences of disability, her/his adaptation, resilience, and the family background contribute defining patient’s quality of life [8]. Glasgow Outcome Score (GOS), its extended form (GOSE) and modified Rankin Scale (mRS) are the most used tools to evaluate disability after SAH, while Short Form (36) Health Survey and EuroQoL are the most used to evaluate the quality of life in this group of patients [9]. The long-term outcomes of SAH patients have been evaluated in several studies [6]. However, to our knowledge, there are no studies that evaluate the relationship between clinical presentation and long-term disability and quality of life in SAH patients admitted to the ICU, a restricted group of patients who often exhibit distinctive outcomes compared to others. Our pilot study aims to describe the long-term outcome and quality of life of patients affected by SAH admitted to our ICU and to establish if there is a relationship between outcome and initial clinical presentation or DCI.

## Methods

This is a single-center, observational, retrospective cohort study. We retrospectively analyzed our electronic clinical records. The study was approved by the Research Ethics Committee of AUSL Romagna (approval number 3041, 11/06/2021). Informed consent was obtained from all the patients. This study adheres to the applicable STROBE guidelines [10].

### Study population

We enrolled all the patients admitted to our ICU from 1st January 2020 to 31st December 2020 with a diagnosis on SAH according to European Guidelines [11].

The inclusion criteria were as follows:

- Diagnosis of SAH (ICD-9-CM Diagnosis Code 430, nontraumatic SAH, unspecified) at admission in the ICU within the period of the study
- Age ≥ 18 years-old at follow-up

Patients in whom neither neurosurgical nor neurointensive treatments had been started because of catastrophic clinical/radiological signs of brain injury were excluded from data analysis.

Patients who were lost to follow-up were not included in the main analysis. Patients that were not directly admitted to ICU after aneurysm exclusion procedure were not included.

### Data collection

The following data were extracted from clinical records:

- Baseline data: age, sex, history of smoking, chronic arterial hypertension, diabetes mellitus, WFNS scale, HH scale, mFS, GCS at admission
- Aneurysm treatment: endovascular vs neurosurgical, requiring of an external ventricular drain (EVD)
- Complication occurred during ICU stay: early (< 24 h) intracranial hypertension, hydrocephalus, late (> 24 h) intracranial hypertension, seizures, central neurological fever, hyponatremia, nimodipine interruption due to hypotension, increased middle cerebral artery (MCA) mean flow velocity, vasospasm location, impaired CT perfusion, induced hypertension therapy, endovascular vasospasm treatment
- Outcome measures: hospital length of stay, 12-month Glasgow Outcome Scale Extended (GOSE), 12-month EuroQoL-5D-3L Index (EQ-5D Index), and EQ-VAS

### General management

Aneurysm exclusion was performed as soon as possible. The type of securing procedure was decided after a multidisciplinary discussion between neuroradiologists, neurosurgeons, and intensivists. EVD was placed in case of acute hydrocephalus, HICP, or for surgical needs. All patients with GCS ≤ 8, delayed awakening after procedure or critical medical condition were admitted to ICU. In all patients, a CT scan was obtained within 24 h after aneurysm exclusion.

### Management of vasospasm and delayed cerebral ischemia

Nimodipine was administered orally 60 mg q4h. If patients showed poor tolerance to nimodipine developing hypotension, nimodipine administration was suspended. Sedation was suspended as soon as possible in all the patients without HICP, delirium, hemodynamic instability, or acute respiratory failure, while TCD monitoring through the acoustic transtemporal window was performed daily to detect ultrasonographic vasospasm [12]. In case of clinical deterioration or ultrasonographic vasospasm, supported by clinical judgment, a CT angiography/CT perfusion or cerebral arteriography was obtained. Patients with vasospasm/DCI were treated with blood pressure augmentation. In case of refractory vasospasm/DCI, endovascular rescue therapy was considered.

Complications were defined as follows:

- High intracranial pressure (HICP): This was defined as intracranial pressure > 20 mmHg sustained for a period > 5 min [13, 14]. If it occurred within 24 h from admission, it was defined early HICP; if it occurred after this period, it was defined as late HICP.
- Seizures: EEG were performed as early as possible in comatose patients. Electrographic and electroclinical seizures, electrographic status epilepticus, and electroclinical status epilepticus were diagnosed according to the current American Clinical Neurophysiology Society guidelines [15]. Seizures were treated with antiepileptic drugs and, in most severe cases, with deep sedation. EEG during sedation holiday was performed to evaluate efficacy of antiepileptic therapy.
- Central neurological fever: central neurologic fever was defined on clinical judgment accordingly to the criteria reported by Hoker et al. [16].
- Hyponatremia: patients requiring sodium implementation to maintain serum level within normal range.
- Symptomatic vasospasm and delayed cerebral ischemia: accordingly with Rass et al., we used the following definition [17, 18]:
  - ◦ Clinical deterioration: New focal neurological deficit OR GCS 2 points OR NIHSS 2 points once excluded other causes (e.g., fever, hyponatremia, hydrocephalus).
  - ◦ Angiographic vasospasm was defined as a reduction in arteries diameter of at least one-third, measured on either CTA or digital subtraction angiography (DSA).
  - ◦ Delayed cerebral infarction (DCIn) was defined as an infarction on CT scan or MRI scans performed within 6 weeks after SAH, absent from the scan performed between 24 and 48 h after aneurysm occlusion, and not attributable to another cause: aneurysm-securing procedure or EVD placement.

### Outcome and telephone interview

Hospital length of stay (H-LOS) was obtained from electronic health-data records. Days of H-LOS were considered from admission to one of these possible outcomes: death, discharge home, and discharge to rehabilitation institutes.

Telephone interviews were performed between 12 and 15 months after initial bleeding by two operators to evaluate outcome. When patients were unable to reply to the survey, the patient’s caregiver was interviewed. Degree of disability and survival were measured using the GOSE [19]. Favorable outcome was defined as GOSE ≥ 4 while patients with GOSE ≤ 3 as unfavorable outcome [20]. Quality of life was evaluated using EQ-5D-3L [21]. The EQ-5D Index scores were calculated from the vectors by using the UK tariff [22]. The nominal range of the EQ-5D Index scores is 0 to 1, but negative scores as low as − 0.59 are possible for health states deemed to be worse than death. The EQ-VAS, instead, records the patient’s self-rated health on a 0–100 scale where 0 represents “the worst health you can imagine,” while 100 is the “best health you can imagine.” When the patients are unable to answer, it is asked to caregivers to give the answer the patient would have given.

### Endpoints

Objectives our study were as follows:

- To describe the long-term outcome of patients admitted to ICU following SAH
- To describe quality of life of patients admitted to ICU following SAH

The secondary objective of the study was to establish if there is a correlation between baseline characteristics, clinical course and outcome.

### Statistical analysis

Statistical analysis was performed using the software IBM SPSS 22.0. Data are reported as mean and standard deviation (SD), median and interquartile range (IQR), and number and percentage (N, %), depending on the underlying distribution.

Independent Student’s *t* test, Mann–Whitney *U* test, Pearson’s chi-squared test, Fisher’s exact test, and Kruskal–Wallis test were used to test any difference between the “favorable” and “unfavorable “groups.

Secondly, to test correlations between different variables, we measured Spearman’s rank correlation coefficient.

Due to small sample size, we did not use binary logistic regression analysis as it not reliable in this situation. A *p* value less than 0.5 is statistically significant.

## Results

### Baseline data

**Fig. 1:**
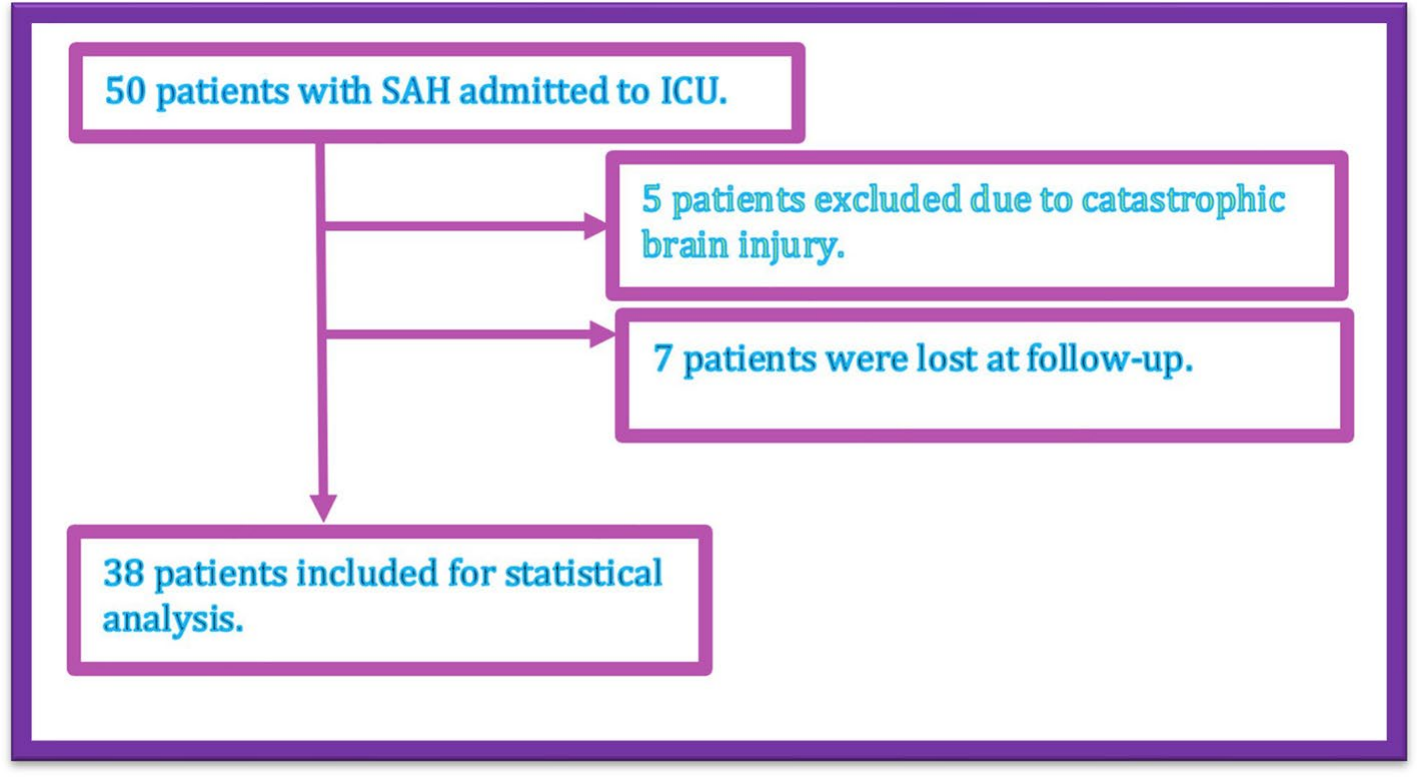
Flowchart for cohort selection

Thirty-eight patients were eligible for statistical analysis (Fig. 1).

Table 1 summarizes main baseline characteristics.

**Table 1:** Relation between baseline characteristics at admission and outcome

			Total	Favorable*	Unfavorable*	*p V*alue	Missing
Female	*N* (%)		24 (63.2)	18 (75.0)	6 (25.0)	0.081	-
Male	*N* (%)		14 (36.8)	6 (42.9)	8 (57.1)		
Current smoking	*N* (%)		11 (28.9)	6 (54.5)	5 (45.5)	0.493	
Chronic hypertension	*N* (%)		21 (55.3)	13 (61.9)	8 (38.1)	0.859	-
Type II diabetes	*N* (%)		2 (5.3)	2 (100)	-	0.267	-
Age	Median (IQR)		58 (20)	53.5 (17)	60 (19)	0.098	-
Glasgow Come Scale	*N* (%)		37	23 (62.1)	14 (37.8)	0.156	1
	Median (IQR)		11 (8.25)	13 (5.5)	6 (11)		
WFNS scale	1	*N* (%)	10 (27.0)	6 (60)	4 (40)	0.078	1
	2	*N *(%)	5 (13.5)	5 (100)	-		
	3	*N* (%)	7 (18.9)	5 (71.4)	2 (28.6)		
	4	*N* (%)	5 (13.5)	4 (80)	1 (20)		
	5	*N* (%)	10 (27.0)	3 (30)	7 (70)		
Hunt-Hess scale	1	*N* (%)	-	-	-	0.033	1
	2	*N* (%)	11 (29.7)	7 (63.6)	4 (26.4)		
	3	*N* (%)	9 (24.3)	9 (100)	-		
	4	*N* (%)	3 (8.1)	1 (33.3)	2 (66.7)		
	5	*N* (%)	14 (37.8)	6 (42.9)	14 (37.8)		
mFisher scale	1	*N* (%)	-	-	-	0.165	-
	2	*N* (%)	6 (15.9)	5 (83.3)	1 (16.7)		
	3	*N* (%)	3 (7.9)	3 (100)	-		
	4	*N* (%)	29 (76.3)	16 (55.2)	13 (44.8)		

*Percentages in the columns “favorable” and “unfavorable” refer to the variable analyzed, not to the overall population

Twenty-four (63.2%) patients were female. Median age was 56 years old (IQR 20). The median GCS on arrival was 11 (IQR 8.25).

There were no age and sex differences between favorable/unfavorable outcome groups. Comorbidities evaluated in our study did not show any difference among the two groups.

One patient had no GCS reported on the record. This patient was included in the primary outcome analysis, but it was excluded in the secondary outcome analysis requiring that data. GCS at admission was lower in the unfavorable outcome group, but it did not show a linear relation with GOSE.

WFNS scale, HH scale and mFS are summarized in Table 1.

In our cohort of patients, only HH scale showed a significant relation with disability at 1 year.

### Treatment

#### Univariate analysis showed no difference between surgical vs endovascular treatment in our population

One patient did not receive any treatment because it was not detected any lesion (SAH *sine materia*).

Twenty-two (57.9%) patients had hydrocephalus at admission. EVD was placed in 23 patients (60.5%). In one patient, EVD was placed not to treat hydrocephalus but for surgical purposes.

### Complications

Table 2 summarizes relation between clinical course in ICU and outcome.

**Table 2:** Relation between clinical course in ICU and outcome

			Total	Favorable*	Unfavorable*	*p* Value	Missing
Treatment	Endovascular	*N* (%)	19 (51.3)	11 (57.9)	8 (42.1)	0.582	1
	Surgical	*N* (%)	18 (48.6)	12 (66.7)	6 (33.3)		
Hydrocephalus		*N* (%)	22 (57.9)	13 (59.1)	9 (40.9)	0.542	-
Neurological fever		*N* (%)	27 (71.0)	18 (66.7)	9 (33.3)	0.482	
Early HICP (<24 h admission)		*N* (%)	11 (28.9)	4 (36.4)	7 (63.6)	0.028	-
Late HICP (>24 h admission)		*N* (%)	10 (26.3)	4 (40)	6 (60)	0.077	-
Hyponatremia		N(%)	18 (47.4)	12 (66.7)	6 (33.3)	0.671	
Seizures		*N* (%)	5 (13.6)	3 (60.0)	2 (40.0)	0.569	-
Nimodipine interruption		*N* (%)	12 (31.6)	9 (75.0)	3 (25.0)	0.309	-
Clinical deterioration		*N* (%)	7 (18.4)	5 (71.4)	2 (28.6)	0.693	-
TCD Vmean MCA > 120 cm/sec		*N* (%)	16 (42.1)	10 (62.5)	6 (37.5)	0.942	-
TCD Vmean MCA > 160 cm/sec		*N* (%)	12 (31.6)	8 (66.7)	4 (33.3)	0.760	-
TCD Vmean MCA > 200 cm/sec		*N* (%)	8 (21.0)	6 (75.0)	2 (25.0)	0.434	-
Vasospasm (MRA/CTA or catheter angiography)		*N* (%)	13 (34.2)	9 (69.2)	4 (30.8)	0.931	
Impaired CT/MR perfusion		*N* (%)	4 (10.5)	2 (50)	2 (50)	0.409	
Hospital length of stay (H-LOS)		Median (IQR)	22 (12)	19.5 (13)	22.5 (8)	0.540	

*Percentages in the columns “favorable” and “unfavorable” refer to the variable analyzed, not to the overall population

Among complications occurred during ICU-stay only early HICP was related with unfavorable outcome (*p* = 0.028).

Eleven (28.9%) patients developed early HICP, while 10 (26.3%) developed late HICP. Intracranial hypertension was treated implementing sedation, with hyperosmolar therapy, CSF withdrawal, normo-hypocapnia, and, in most extreme cases, surgical decompression.

### Vasospasm and DCI

**Fig. 2:**
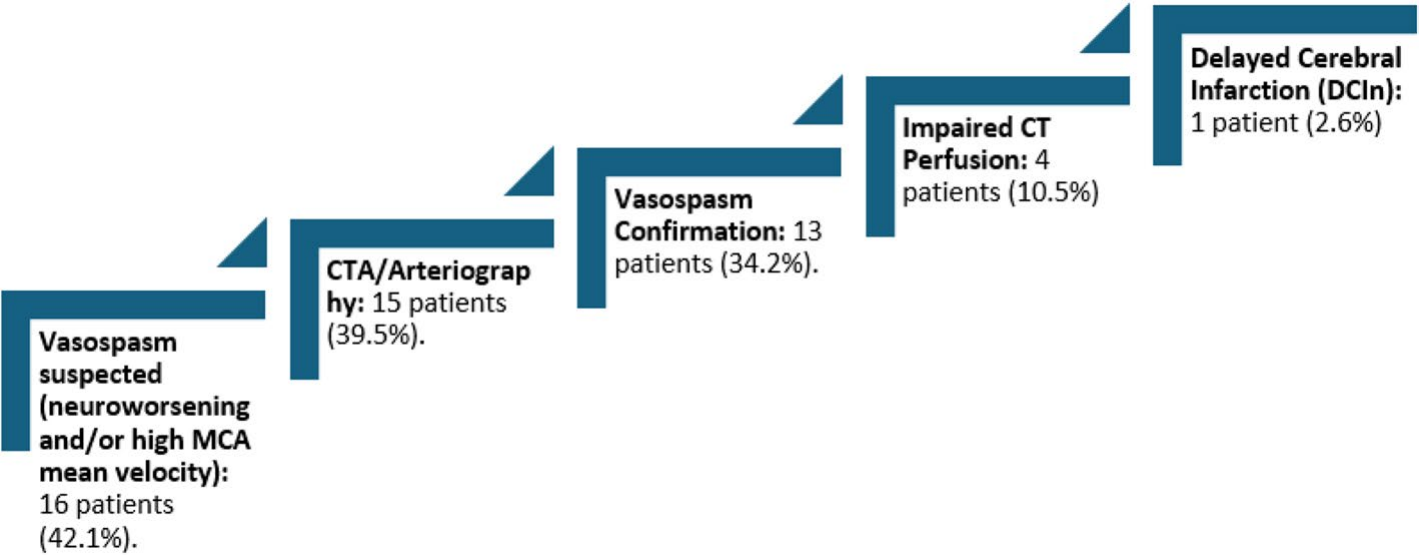
Vasospasm and delayed cerebral ischemia detection

Figure 2 summarizes incidence of vasospasm and DCI. Seven patients (18.4%) showed clinical deterioration defined as new focal neurological deficit or loss of 2 points on GCS or 2 points on NIHSS. All of them had an increase in MCA mean flow velocities of at least 120 cm/sec.

Of the 16 patients with MCA mean flow velocities > 120 cm/s, CT angiography and/or arteriography were performed in fifteen patients (39.5%). Vasospasm was confirmed on CTA/arteriography in thirteen patients (34.2%). Twelve patients (31.6%) required induced therapeutic hypertension. Nimodipine was interrupted in twelve patients (31.6%) due to poor tolerance.

Only four patients (10.5%) showed impaired perfusion on CT scan. Of the four patients with impaired CT perfusion, three patients underwent endovascular rescue treatment. Only one patient, the one with impaired CT perfusion who did not undergo to endovascular rescue therapy, developed delayed cerebral infarction (DCIn).

### Outcome

Median H-LOS was 22 days (IQR 12), and we did not find any significantly differences among the two groups [19.5 (IQR 13) vs 22.5 (IQR 8), *p* = 0.540].

Long-term neurological outcome of the 38 patients is displayed on Table 3. Twenty-four patients (63.2%) had favorable outcome (GOSE ≥ 4). GOSE at 1 year is summarized on Table 3.

**Table 3:** GOSE at one year

	GOSE at 1 year	*N* (%)
**Unfavorable outcome**	1	9 (23.7)
	2	1 (2.6)
	3	4 (10.5)
**Favorable outcome**	4	5 (13.2)
	5	1 (2.6)
	6	5 (13.3)
	7	8 (21.0)
	8	5 (13.2)

Quality of life was evaluated among patients who were alive at 1 year.

Among 29 patients (76.3%) survived at 1 year, median EQ-5D Index was 0.743 (IQR 0.287), while median EQ-VAS was 74.79 (IQR 18.5). Median EQ-5D Index and median EQ-VAS were higher among patients with favorable outcome [EQ-5D Index 0.796 (IQR 0.2) vs − 0.331 (IQR 1.0) *p* = 0.037], [EQ-VAS 80 (IQR 20) vs 50 (IQR 37.5) *p* = 0.003].

**Fig. 3:**
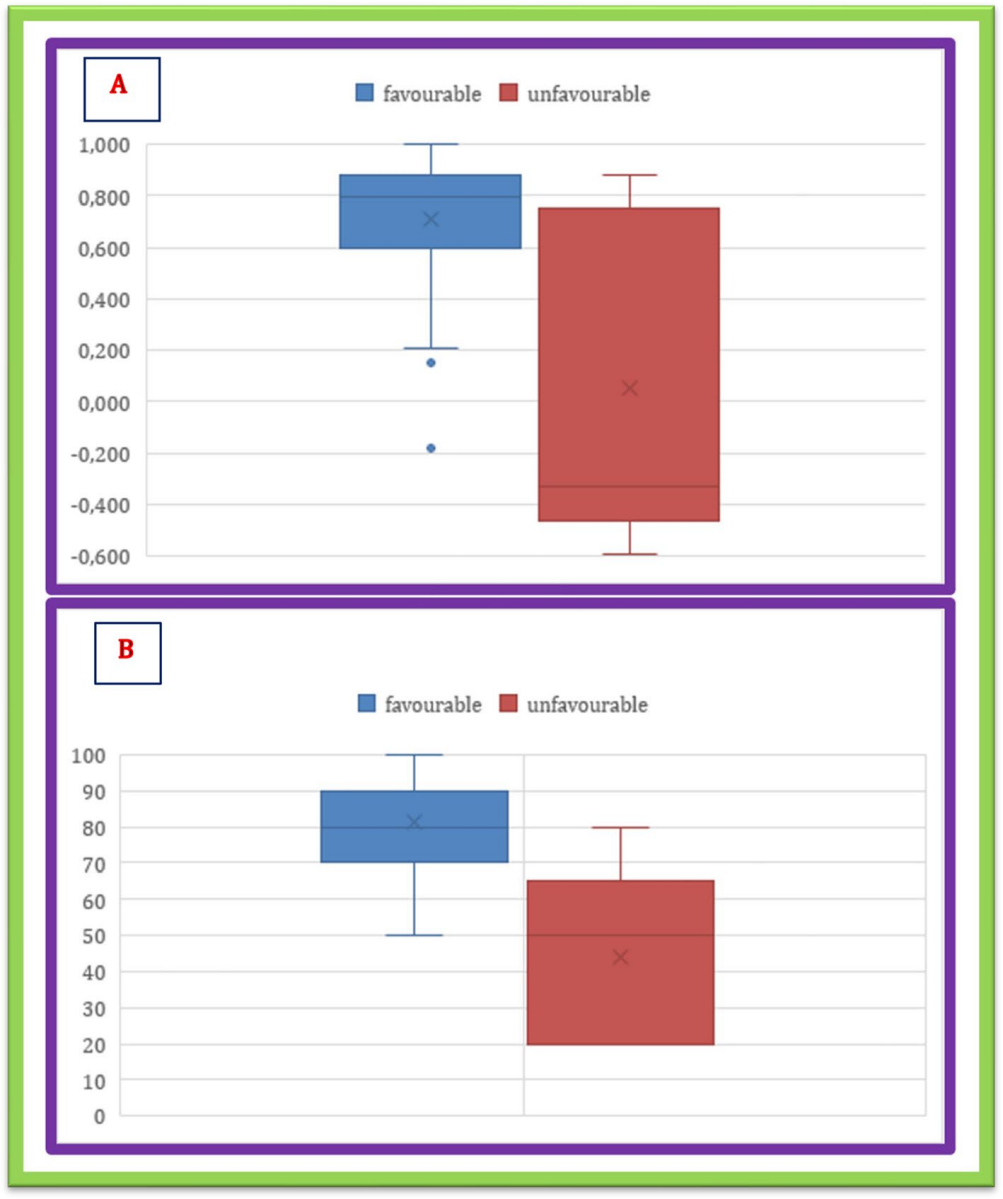
Distribution of EQOL-Index (**a**) and EQOL-VAS (**b**) in the group with favorable (blue) and unfavorable (red) outcome

Relations between GOSE and EQ-5D Index and EQ-VAS are shown in Table 4 and Fig. 3.

**Table 4:** Relation between GOSE and EQ-Index

**GOSEEQ-VAS**				
**2**	− 0.331 (0)	*p* = 0.051	20 (0)	*p* = 0.019
**3**	0.144 (1.2)		50 (30)	
**4**	0.587 (0.45)		70 (7.5)	
**5**	0.587 (0)		96 (0)	
**6**	0.812 (0.38)		85 (18.75)	
**7**	0.83 (0.18)		80 (15)	
**8**	0.812 (0.22)		88 (16.25)	

Data are reported as median and interquartile range (IQR)

## Discussion

To our knowledge, this is the first study that describes long-term disability and quality of life of SAH patients admitted to ICU as primary outcome.

As highlighted by several papers, patient-centered outcomes are key elements in clinical studies [23, 24].

Regarding disability, the study of Chalard et al. is the only one that evaluates long-term disability in a cohort of SAH patients admitted to ICU. This study showed a higher incidence of poor outcome compared to our cohort [6].

There are some reasons that could explain these findings: First of all, we used a different scale to evaluate disability compared with the study reported above. We used GOSE rather than mRS because in our center is the most used scale to evaluate long-term outcome in patients with acute brain injury and, with GOS, it has been widely used in literature in SAH patients [9].

Secondly, we excluded patients with catastrophic brain injury in which treatment was considered futile since admission.

Finally, our higher incidence of good outcome could be related to a low cut-point on GOSE. There is no consensus on the cut-off point to define favorable outcome with GOSE in acute brain injury patients. As shown in a recent paper by Zuckerman et al. [25] there is a wide range of cut-points on GOSE in patients with acute brain injury that ranges from 3 [26] to 7 [27]. Accordingly with the other authors [20, 28], we included GOSE 4 among favorable outcome, recognizing functional independence for at least 8 h as a favorable outcome for patients and caregivers.

Our results seem to support our choice; as shown in Table 3, EQ-5D Index and EQ-VAS in group GOSE 4 are more similar to higher grades (GOSE 6–8) than to the lower ones.

Another major issue of discussion regarding outcome is the quality of life perceived by the patients. Median EQ-VAS varies significantly among GOSE grades (*p* = 0.019), while median EQ-index is not significantly different. This could be related to a small sample-size, but also to a broader effectiveness of EQ-VAS in summarizing overall health that is closer to the patient’s perspective. As reported in Table 3, the highest GOSE grades are not the ones with the highest values of EQ-5D Index and EQ-VAS. This is a phenomenon known as disability paradox [29]. Several studies have shown that many patients, despite a severe disability after brain injury, enjoy a high quality of life [30]. It is remarkable the broad range of EQ-VAS and EQ-5D Index in the GOSE 3 group in our cohort: In fact, in this group usually considered an unfavorable outcome in studies on acute brain injury, there are a patient with poor quality of life (EQ-5D Index -0.594 and EQ-VAS of 20) and a patient with good quality of life (EQ-5D Index 0.883, EQ-VAS of 80). Should this still be considered a poor outcome, or should we focus our attention on the perceived quality of life? It is a thought question that goes behind the purpose of this study, but that should foster a debate.

This poses, if possible, even major challenges to physicians when talking about prognostication in patients with poor-grade SAH and highlights the importance of shared-decision making with the patients and/or the family in this field [31]; moreover, this remarks the importance of patient-centered outcome studies.

Unfortunately, few are the tools available during ICU stay to evaluate prognosis in this group of patients.

Regarding baseline data, in our cohort, females seem to have a better prognosis compared to males; to our knowledge, in previous reports are reported no sex differences in long-term outcome of SAH patients [32]. This could be related to a small sample size and it will require further studies to understand this finding.

In this study, of the most used severity scale, only HH scale seems to show a relation with poor outcome. This scale, introduced in 1968, was first used to predict the rate of mortality based solely on the clinical features in SAH patients; higher HH grades have been associated with poor outcomes also in more recent studies [33, 34]. However, since HH scale does not take in account the presence of reversible causes of coma such as hydrocephalus and seizures, we agree that this scale should not be used alone to define the prognosis of SAH patients.

Consistently with other studies [6, 35], GCS at admission is related to long-term outcome in our cohort. The level of consciousness in patients admitted with SAH could represent the epiphenomenon of the early brain injury that is developing after aneurismal rupture; the pathophysiology of this process is not completely understood and should be the object of further studies. Nonetheless, a poor GCS at admission could be associated with a good recovery as shown by Hoogmeoed et al. [36].

Among SAH-related complications, only high early intracranial hypertension was related with poor outcome in our cohort. This finding could have different explanations. Early HICP could be related to a well-known cause such as hydrocephalus, but this was not related to poor outcome in our cohort. Possibly, early HICP could be a surrogate marker of EBI due to loss of autoregulation [37] or cerebral edema [14, 38].

As highlighted in other papers, our data support the lack of relation between vasospasm detected on TCD and/or CTA and long-term outcome. The incidence of vasospasm is similar to other cohorts of patients [6] and emphasizes the importance of distinguishing between vasospasm, DCI and DCIn [4]. As highlighted by recent papers, DCI has a complex pathology in which vasospasm is just one of the determinants of brain injury. TCD and angiographic spasm seem to pose the patients at higher risk of DCI, but this is not related to long-term outcome [39–41].

Instead, DCI can lead to DCIn, a well-known factor related to long-term poor outcome.

DCI can be suspected in patients with clinical deterioration or in patients with impaired CT-perfusion [17]. As remarked by the same authors that proposed the definition of clinical deterioration due to DCI in 2010, diagnosis of DCI can be tricky due to several confounders, especially in ICU patients [18]. The presence of several confounders combined with the small sample size could explain the lack of relation between DCI and long-term outcome in our cohort. Another possible explanation is that DCI is not strictly related to DCIn. In fact, in our cohort, only 25% of patients who had impaired CT-perfusion developed DCIn. This seems to support the hypothesis that DCI detected on CT-perfusion still represents a reversible situation that requires the highest quality of care to prevent DCIn [42, 43].

## Limitations

The main limitations of the study are the small sample size and the fact that it is a single center study. A larger number of cases would allow multivariate analysis to identify independent outcomes predictors.

mRS seems to be a better outcome measure for SAH patients, but GOSE is still widely used. Moreover, in our institution, GOSE is widely used as an outcome scale for patients with acute brain injury, and physicians and nurses are well-trained in its use.

Regarding dichotomizations, there are several cut-offs reported in the literature that could limit the comparison between studies.

Another limitation is the use of caregivers as surrogate to evaluate QoL, but this is recommended in all the cases in which the patients it is unable to answer. This study does not include patients admitted in good neurological condition and managed in the neurosurgery ward throughout their hospital stay.

## Conclusions

This study describes long-term disability and quality of life in SAH patients admitted to ICU and their relation with clinical features. High HH grades and early HICP were related with unfavorable outcome. Among patients with unfavorable outcome, quality of life has a broad range of variability, and this result should be taken into account when reporting patient-centered outcomes.

## Data Availability

The datasets generated during and/or analysed during the current study are available from the corresponding author on reasonable request.

## Abbreviations

SAH: Subarachnoid hemorrhage
DCI: Delayed cerebral ischemia
DCIn: Delayed cerebral infarction
EBI: Early brain injury
ICP: Intracranial pressure
HICP: High intracranial pressure
ICU: Intensive Care Unit
EVD: External ventricular drain
GOSE: Glasgow Outcome Scale–Extended
mRS: Modified Rankin Scale
Hunt-Hess Scale: HH Scale
mFS: Modified Fisher Scale
WFNS: World Federation of Neurological Surgeons
TCD: Transcranial doppler
