# Correction: One-year outcome and quality of life of patients with subarachnoid hemorrhage admitted to intensive care unit: a single-center retrospective pilot study

**DOI:** 10.1186/s44158-025-00230-5

**Published:** 2025-02-20

**Authors:** Carlo Bergamini, Etrusca Brogi, Sara Salvigni, Cesena Outcome Group, Michele Romoli, Giovanni Bini, Alessandra Venditto, Elvis Lafe, Marcello D’Andrea, Luigino Tosatto, Maria Ruggiero, Vanni Agnoletti, Emanuele Russo

**Affiliations:** 1 Department of Emergency Surgery and Trauma, Anesthesia and Intensive Care Unit, Bufalini Hospital, Azienda Unità Sanitaria Locale (AUSL) Della Romagna, Cesena, Italy; 2 Neuroscience Intensive Care Unit, ASST Grande Ospedale Metropolitano Niguarda, Milan, Italy; 3 Neurology and Stroke Unit, Department of Neuroscience, Bufalini Hospital, Cesena, Italy; 4 Neuroradiology, Department of Neuroscience, Bufalini Hospital, Cesena, Italy; 5 Department of Neurosurgery, Maurizio Bufalini Hospital, Cesena, Italy

**Correction: J Anesth Analg Crit Care 5, 2 (2025)**

**https://doi.org/10.1186/s44158-024-00223-w**

In the original publication of this article [1] the author names were transposed. The incorrect and correct information is shown in this correction article, the original article has been updated.

Incorrect

Bergamini Carlo, Brogi Etrusca, Salvigni Sara, Cesena Outcome Group, Romoli Michele, Bini Giovanni, Venditto Alessandra, Lafe Elvis, D’Andrea Marcello, Tosatto Luigino, Ruggiero Maria, Agnoletti Vanni & Russo Emanuele

Correct

Carlo Bergamini, Etrusca Brogi, Sara Salvigni, Cesena Outcome Group, Michele Romoli, Giovanni Bini, Alessandra Venditto, Elvis Lafe, Marcello D’Andrea, Luigino Tosatto, Maria Ruggiero, Vanni Agnoletti & Emanuele Russo
